# Adherence and Uptake of Artemisinin-Based Combination Treatments for Uncomplicated Malaria: A Qualitative Study in Northern Ghana

**DOI:** 10.1371/journal.pone.0116856

**Published:** 2015-02-18

**Authors:** Samuel Chatio, Raymond Aborigo, Philip Baba Adongo, Thomas Anyorigiya, Patricia Akweongo, Abraham Oduro

**Affiliations:** 1 Navrongo Health Research Centre, P.O Box 114, Navrongo, Ghana; 2 University of Ghana, School of Public Health, Accra, Ghana; Swiss Tropical & Public Health Institute, SWITZERLAND

## Abstract

**Background:**

Based on the recommendations of the World Health Organization in 2004, Ghana changed her antimalarial drug policy from mono-therapy to Artemisinin-based Combination Therapy (ACTs). The country is currently using three first line drugs artesunate-amodiaquine, artemether-lumefantrine and dihydroartemisinin-piperaquine for the treatment of uncomplicated malaria. Despite this policy, little or no qualitative studies have been conducted to establish the factors influencing adherence to the new treatment for malaria. This study explored factors influencing adherence to the use of ACTs in northern Ghana.

**Methods:**

This was a qualitative study comprising forty (40) in-depth interviews with patients with malaria who visited selected public and private health facilities and received ACTs. Systematic sampling technique was used to select participants who were given ACTs for the interviews. Nvivo 9 software was used to code the data into themes for further analysis.

**Results:**

The study revealed very important differences in knowledge about ACTs. As expected, the less or illiterates could not mention the type of ACT they would prefer to use for treating their malaria. The educated ones had a good knowledge on ACTs and preferred artemether-lumefantrinee in treating their malaria. The reason was that the drug was good and it had minimal or no side effects. Individual attitudes toward the use of medications and the side effects associated with the use of these ACTs were found to be the main factors affecting adherence to the use of ACTs. Perceived cure of illness after the initial dose greatly affected adherence. Other factors such as forgetfulness and lack of information also influenced patient adherence to ACTs use.

**Conclusion:**

Individual knowledge, attitudes and behaviors greatly influence patients’ adherence to ACTs use. Since ACTs take a number of days to complete, continuous education by health professionals could improve on adherence to ACTs use by patients with malaria.

## Introduction

According to the World Health Organization, malaria remains a major public health problem causing about 207 million cases with 627,000 deaths in 2012 mainly in sub- Saharan Africa [1]. The most vulnerable groups are children under five years and pregnant women [2]. Several interventions such as Intermittent Preventive Treatment of Malaria in Infants (IPTi) and Pregnant Women (IPTp), Indoor Residual Spraying (IRS), the use of Insecticide Treated Nets (ITNs) and lately Seasonal Malaria Chemoprophylaxis in children under five years (SMC) have been introduced to help reduce the burden of malaria particularly in Africa [3, 4].

Despite these interventions, malaria remains endemic in Ghana and is the single most important cause of morbidity and mortality especially among young children, pregnant women and the poor [5]. About 3.5 million clinical cases are recorded in Ghana every year with about 20,000 deaths occurring in children under five years of age [2]. In 2011, malaria accounted for about 38% of all outpatient illnesses, 36% of all admissions and 33% of all deaths among children under five years of age [3]. The annual economic burden of malaria in Ghana is estimated to be 6 per cent of the Gross Domestic Product [2, 5].

In early 2000, the World Health Organization (WHO) recommended to all countries experiencing resistance to mono-therapies to use artemisinin-based combination treatments (ACTs) in treating uncomplicated *falciparum* malaria [6]. Based on the recommendations couple with other factors such as efficacy, cost effectiveness, local industry capacity and some demographic reasons such as the appropriateness for treating in children under five years and in pregnancy, different ACTs were selected as first line drugs to replace the existing mono-therapeutic drugs [7, 8].

Countries with high malaria burden need robust information to inform policy decisions about the benefits of wider implementation of new products. Large scale studies to determine the effectiveness of new drugs when delivered through real-life settings are therefore necessary. For instance, data on patients’ adherence and factors affecting the use of new anti-malaria drugs when delivered outside trial conditions are needed to inform policy decisions. The INDEPTH Network Effectiveness and Safety Study (INESS) platform was therefore established to providing effectiveness data to inform policy direction [9].

Adherence refers to the extent to which patients use medications as prescribed by health providers and is an important component of infectious disease control [10, 11]. For the newly introduced ACTs, various factors may account for non-adherence to their use in real life settings. Some studies have reported that about seventy-six percent (76%) of patients with malaria failed to complete their treatment as prescribed due to poor knowledge on malaria [10]. It has also been established that some people would usually use medications partially or stop the treatment once the symptoms subside and keep the remainder to be used in future [12, 13]. Forgetfulness and poor relationship between health professionals and patients have been reported to affect adherence to the use of medications including ACTs [11, 12, 14]. The use of complex or technical terminologies by prescribers has also been reported to influence adherence to the use of medications [15].

In 2004, Ghana changed the national anti-malarial drug policy recommending the use of artesunate-amodiaquine combination as the first line drug for treating uncomplicated malaria. However, the implementation process was faced with several challenges related to side effects from the use of these drugs and lack of other treatment options [5]. It therefore became necessary to review the new policy to address all recognized concerns. The existing policy was reviewed by a task force which selected additional ACTs and dosage forms to cater for those who could not tolerate the earlier artesunate-amodiaquine combination. Consequently, two additional first line ACTs, artemether-lumefantrine and dihydroartemisinin-piperaquine have also been introduced. [5].

These medications have since been in use for about a decade now. However, little is known about the factors influencing the use and adherence to these treatments. This study was therefore designed to explore the factors influencing adherence to the use of ACTs in treating uncomplicated malaria in real-life settings following the introduction of the three ACTs as first line treatments for uncomplicated malaria in Ghana.

## Methods

### Ethical consideration

Ethical approval for the study was received from the Navrongo Health Research Centre Institutional Review Board (NHRCIRB 152). The board emphasized on the need to maintain confidentiality of Participants’ information. In line with the approved procedure of obtaining consent for the study, oral consent was obtained from each potential participant prior to being interviewed and this was approved as part of the protocol for this study. Oral consent was solicited and obtained as the majority of the respondents had no formal education and those with formal education also opted for this method of consent.

The interview moderators read and translated the consent form into the preferred local language of each participant on the purpose of the study, study procedure, right to withdraw and efforts to ensure confidentiality. The oral consenting process was recorded on a separate voice recorder from the one used for the actual interview. In addition, they were made to recommend a member of the household to witness the consenting process and the demographic data of the witnesses were collected. All children from 10 to 17 years old gave assent while their parents/caretakers gave consent before the interviews were conducted.

### Study site

The study was conducted in the Kassena-Nankana East and West Districts by the Navrongo Health Research Centre. The research centre operates the Navrongo Health and Demographic Surveillance System (NHDSS) in the two districts. The districts cover an area of 1,675 square kilometres of Sahelian savannah with a population of about 153,000 [16]. The main languages spoken in the two districts are Kasem and Nankani. The population is predominantly rural with subsistence farming as the mainstay of the districts’ economy. People live in multi-household compounds in the two districts. There are two distinct seasons; the rainy season which spans from May to October with the rest of the year being dry. The malaria burden in the districts is seasonal with the high transmission period occurring between June to October coinciding with the rainy season [17, 18, 19].

The districts have one hospital, eight health centers, two private clinics, which provide curative and preventive health care services to community members. There are 28 Community-based Health Planning and Services (CHPS) compounds located in various communities and providing reproductive health services and treatment of minor health conditions [20, 21]. The district hospital located in Navrongo Township serves as a referral point for all health facilities operating in the two districts. There is one pharmacy shop and over 50 drug and chemical shops.

### Sampling of health facilities

The two districts have been demarcated into five zones by the NHDSS [16]. Two zones representing each of the two dominant languages in the districts Kasem (northern zone) and Nankani (southern zone) were randomly selected for the study. This was to elicit views on any cultural influence on adherence to ACTs. The two health centres located in the selected zones were used for the recruitment of study participants. War Memorial hospital and the only pharmacy shop located in central Navrongo were also purposively selected as recruitment sites of study participants. The facilities were selected due to their high patronage for malaria treatment in the two districts. The District Mutual Health Insurance Scheme has ten accredited chemical shops located in the two districts. The only two accredited chemical shops, one located in each zone were used as recruitment centres.

### Sampling of respondents

All patients who visited and received an ACT in the selected health facilities/chemical shops qualified to participate in the study. This was to enable patients share their experience on the use of ACTs that they received. The data collectors spent two days in each of the six study health facilities/chemical shops, systematically selecting every second patient who was given an ACT. The longest duration for taking ACTs is three days. All recruited participants were therefore followed-up on the fourth day when all of them were expected to have completed their doses. The longest period for the interviews after treatment was three days after the last dose was supposed to have been taken. In all, 40 interviews were conducted in the six health facilities/chemical shops. The themes explored included preference of ACTs in the management of malaria, factors responsible for non-adherence to the use of ACTs and suggested ways to address the issue of non-adherence.

### Data collection

In-depth interviews were conducted with individuals who were given ACTs at the selected health facilities/chemical shops. Addresses of potential participants were obtained to enable the researchers trace them for the interviews at home after day three when they were expected to have completed the course of treatment. A total of forty (40) in-depth interviews were conducted with patients with malaria who were given ACTs. The interviews were tape recorded with the consent of participants. Notes were taken by the moderator to serve as backup in case the recording was not done properly. Each interview lasted for about 40 minutes. The interviews were conducted in Kassem, Nankani and English depending on the participant’s preference.

### Training of interviewers

Two university graduates with experience in conducting qualitative interviews were recruited and trained for one week. They were given an overview of the study and trained on consent procedures, interviewing, probing and transcribing of interviews. Role plays were done during the training session where trainees interviewed each other in both English and the local languages and received feedback from the researchers. A pretest was conducted to evaluate their performance and help finalize the interview guide before the actual data collection.

### Data processing and analysis

The recorded interviews were transcribed verbatim and entered into a computer using Microsoft word. Guided by the objectives of the study and the themes contained in the guide, a coding list was prepared for data analysis. The data analysis was initiated simultaneously with data collection. This was to ensure that new themes were incorporated into the guide and that thematic saturation was monitored. The data was organized using QSR Nvivo 9 software before analysis. A codebook was developed based on the major themes of the study and transformed into tree nodes and free nodes. Free nodes are generally referred to as open standalone codes usually used at the beginning of the data coding process and are non-hierarchical in nature. Tree nodes are however, hierarchical in nature and they can have relationships with other nodes. They are usually used for secondary coding after open coding from the first round of data coding. The sampling approach was to enable us elicit ethnic differences in the use of ACTs by the two ethnic groups, Kasem and Nankani. However, the analysis did not show any pattern of differences and so this theme was not considered in the presentation of the results. The results of the study were then based on the major and sub-themes that emerged from the study.

## Results

### Background information of respondents

The age of respondents was grouped into four categories. Most of the respondents, sixteen were between 31–40 years while only four of them were between 41 years and above. The level of education of the respondents was also grouped into three, those who had never attended school, primary to junior high level and secondary to tertiary level. From the table, twenty-one respondents had between primary to junior high education while only six (6) of them had secondary to tertiary education (Table 1).

**Table 1:** Background characteristics of respondents.

**Category**	**Frequency**
Age	
10–20	5
21–30	15
31–40	16
41+	4
	
**Sex**	
Male	9
Female	31
	
**Ethnicity**	
Kasem	22
Nankani	15
Other	3
	
**Education**	
No education	13
Primary/Junior high level	21
Secondary/tertiary level	6

### Knowledge on type of ACT received by patients at the health facility

Our findings revealed that there was vast variation of knowledge about ACTs among the educated and uneducated participants. As expected, most of the uneducated participants had little or no knowledge on the type of ACT they were given. They could not mention the names of any of the three ACTs currently being used to treat uncomplicated malaria as captured below by study participants.

*“Well, I do not know the name because when I went they gave me some medicine that has a mosquito on the pack”*

***(IDI-25 year old female patients).***

*“I wouldn’t be able to tell you the name of the medicine they gave me”*

***(IDI-39 year old male patient).***

However, the educated participants were able to mention the names of the ACT they were given at the health facility or chemical shop. Others could not mention the name but were able to describe the color of the ACT given. White and yellow is commonly used by community members to describe artesunate—amodiaquine. Most of the educated participants knew artesunate-amodiaquine.

*“They gave me the artesunate-amodiaquine…”*

***(IDI-28 year old female patient).***

*“The doctor gave me artemether-lumefantrine at the health facility”*

***(IDI-32 year old male patient)***

*“It is the yellow and white medicine that was given to me”*

***(IDI-27 year old female patient)***

### Preferred ACT for the management of uncomplicated malaria

Diverse views were expressed by educated participants on the ACT they would mostly prefer using to treat their malaria. However, the results showed that artemether-lumefantrine was reported by participants as the most preferred ACT used to treat uncomplicated malaria. Twenty-six respondents reported preference for artemether-lumefantrine for the treatment of malaria. Though, eight of these patients received artesunate-amodiaquine at their last malaria episode but reported that they would have preferred artemether-lumefantrine. The main reason participants gave for their choice of artemether-lumefantrine was that the drug was good and it had minimal or no side effects as compared to the other ACTs especially the artesunate-amodiaquine. Participants expressed their views this way on the most preferred ACT for treating uncomplicated malaria:

*“I prefer the artemether-lumefantrine because it is good for me. I took the artesunate-amodiaquine some time ago and I nearly died. My feet and hands were all stiff, I could not feel anything, I could not open my eyes and I finally ended in the hospital”*

***(IDI-26 year old female patients).***

*“I was given the artemether-lumefantrine; and it is that drug I prefer using anytime I have malaria. For artesunate-amodiaquine no, no, no, I do not like taking it because when I take it, it is like I have added another illness to the one I already have. For artemether-lumefantrine when I took it I was fine because it did not disturb me at all and it is also good”*

***(IDI-31year old female patient).***

Almost all respondents who completed their treatment using artesunate-amodiaquine reported feeling well on the day of the interview. However, participants who did not complete the drug regimen for artesunate-amodiaquine attributed it to the side effects such as body weakness, dizziness, vomiting and loss of appetite. They however, acknowledged that the drug was very good in treating malaria.

*“There are two things, though, the artesunate-amodiaquine that I said I got serious side effects when I took it, since then I have never had malaria up to date…. So I think when you force and take artesunate-amodiaquine it will take you long before you get malaria again….”*

***(IDI-44 year old female patients).***

*“The one I said is white and yellow (refers to artesunate-amodiaquine), I was given that medicine and after taking them I did not want to hear the scent (smell it) again. After taking the full dose, I felt very weak and could hardly hold an object. After a while, I saw that my situation had improved so the issue is that the drug has side effects but when you persevere and take it to the end (take the full dosage) you will be fine”*

***(IDI-26 year old female patient)***

Some of the uneducated participants on the other hand reported that they had no choice on the type of ACT they would prefer using to treat their malaria. They said that since they did not have knowledge on these ACTs or know the names of these ACTs, they had no problem using any of them that was given at the health facility. A 35 year old female patient had this to say:

*“I don’t know their names and I take any malaria drug that is given to me to treat my illness”*

***(IDI-35 year old female patient).***

### Factors responsible for non-adherence to ACT treatments by patients with malaria

Sixteen respondents reported having difficulty completing their malaria doses. Perceived cure of malaria after initial dose affected adherence. According to the participants, they felt that their illness or condition was better after they had taken the initial dose of the medication and that made them to stop taking the remaining medicine. Respondents said this was the core reason why they could not complete the course of treatment.

*“When you take and you feel better, you stop taking the drugs. I will use myself as an example because when I took it I realized it was better and I had to stop taking the other tablets”*

***(IDI-35 year old female patient).***

*“When I took the malaria drug they gave me at the hospital for the first and second days, I felt better and so I had to stop. I don’t usually like taking drugs and because of that when I manage and take them small and realize that my illness is better I stop taking the rest*

***(IDI-46 year old female patients).***

The side effects patients experience from using ACTs also greatly affected adherence to malaria treatments especially with respect to artesunate-amodiaquine. Nine out of fifteen patients who were given artesunate-amodiaquine reported that they had to stop using the drug half way during the treatment when they started experiencing side effects such as body weakness, dizziness and loss of appetite. Respondents reported that artesunate-amodiaquine unlike the other ACTs was such that when you take it, your condition would become rather more serious than before because of the side effects and that accounted for their inability to complete the treatment.

*“It is because some malaria medicines make people weak when you take them; so after taking the first one and getting weak, you get discouraged. Then you feel you should wait till tomorrow and eventually you stop taking it. That was what I did with the artesunate-amodiaquine they gave me at the hospital. I was weak so I had to stop taking it”*

***(IDI-42 year old female patients)***

*“The reason for this is because of the side effects people get from using these drugs. I was given the yellow and white (artesunate-amodiaquine) drug and when I was taking the medicine, I could not cook and because I do not have somebody to do it for me I had to stop taking it. These are some of the reasons why we are sometimes not able to take the drugs.”*

***(IDI-35 year old female patient)***

The problem of recall was also reported in this study as a factor affecting adherence to ACTs use. Participants reported that forgetfulness was a factor responsible for non-adherence to the use of ACTs especially the older people because of the number of times one needed to take the drug in a day and the time they were supposed to take it. A female participant had this to say:

*Q: Did you take the ACT as prescribed to you?*

*R: No, I could not take all. Laughter by respondent and moderator*

*Q: Why were you not able to take all of it?*

*R: I did some work and became tired and I forgot and went to bed. I took it the following day*

***(IDI-male patients)***

The requirement for patients to eat before taking medications including malaria drugs affected adherence. The study found that the inability to eat heavy food in the morning before taking the medications also affected adherence to ACT use by patients with malaria. The taste of some ACTs (being bitter) greatly influenced their proper use by some study participants. A female respondent shared her views this way on the issue:

*“They said I should take it in the morning and evening but I was always taking it only in the evenings. The reason is that I don’t eat heavy food in the morning because they told me to eat heavy food before taking the drugs and once I cannot eat heavy food in the morning was the reason why I was not taking the drug in the morning. Some people because of the “bitterness” (taste) of some of the drugs, they do not want to take it.”*

***(IDI-39 year old female patient).***

Other factors mentioned by participants that affected adherence were lack of knowledge as a result of health providers’ inability to educate patients on the proper use of the medication. Twenty-nine respondents mentioned this as one of the factors affecting ACT use. They reported that they were not properly educated by health providers on how they could use the ACT given. Lack of education was mentioned by both educated and non-educated respondents in this study. Peer influence was also reported by study participants. Respondents reported that patients with malaria were not educated by health providers on how to properly use these ACTs. They said that wrong information received from friends and other relations led to wrong use of ACTs. In the exact words of some of the respondents:

*“It is ignorance and sometimes influence from peers. Yeah, some it is due to peer influence because we don’t consider our individual body system when it come to the use of medicine but one may just come and tell you that as for this drug when they prescribed it to me, I took it this and that way, even a drug like paracetamol some may take the whole sachet when you are only asked to take two tablets when you have headache. Some come and tell you that this is how I took it so also take the same especially if the illness is critical. So, I think it is the peer influence”*

***(IDI-36 year old male patient)***

*“Sometimes when there are so many patients, the health workers do not have patience and because of that they do not take their time to explain how people should take their medications. They are also not able to let them know that if they do not take the medicine according to instructions, it will not work. The health workers usually want the crowd to disperse to enable them go home. So when a patient is told to take twice a day, when he takes it in the morning within a short period, the person will take it again”*

***(IDI-28 year old female patient)***

Some speculative factors affecting adherence were also mentioned by participants. Study participants blamed health providers for patients’ inability to use ACTs according to prescription. They were of the view that advices given by some health providers on the use of ACTs were not accurate and that contributed greatly to non-adherence. They said that was the reason why some people would usually ignore certain prescriptions given by health providers and rather use the drugs their own ways. Participants were of the opinion that people would usually want their illness to heal very fast and as a result they would take over dose of the medication in order to help them in that regard. These factors according to participants were also responsible for malaria patients’ inability to adhere to the use of ACTs.

*“Others feel that the advices given at the hospital are not good and that is why they usually ignore what the doctor told them to take the drugs and instead use it the way they want. Though I have not done that but I know of somebody who said that she had to use her own knowledge to take the drug because the way the health worker asked her to take was not correct”*

***(IDI-44 year old female patient)***

*“For some people they have the motive that when they take the drugs more (overdose) it will help to cure the illness faster. So when the doctor says they should take two in the morning and two in the evening they will rather take three, three with the motive that it will cure the illness faster….”*

***(IDI-39 year old male patient).***

### Suggested ways to address non-adherence to ACTs use

Despite the fact that there are various factors affecting adherence to the use of ACTs, our study participants proposed various strategies to help address the problem. Thirty-five of the respondents said that there was the need for vigorous continuous education on the use of ACTs at the Out Patient Department (OPD) level, the pharmacy department and at the chemical shops by health providers and chemical shop owners. They were of the view that the education should include the need for people to strictly follow dosage prescriptions and use malaria medications and the likely side effects people could get as a result of wrong use of these ACTs at home.

*“The pharmacists and the doctors should do the education; because most of the people here are illiterates they should have time to explain to them how they can take the drugs and why they have to follow prescriptions and take the drugs. We the patients too when they give us the drugs, we should try and take the drugs according to how we have been asked to take…”*

***(IDI-39 year old male patient).***

*“At the OPD when patients are seated and doctors have not started consulting, a nurse should give a talk to all patients on how they should take their medicines. Sometimes when they write three times on the envelop, someone can take it four times. The talk will make patients understand why they should take medicines according to the way they have been instructed”*

***(IDI-38 year old female patient).***

Thirteen respondents suggested a single dose treatment for malaria in order to enhance adherence to ACTs use. This suggestion was made because some people do not usually like taking medications because of the taste, the number of times and tablets they have to take in a day and if the single dose treatment had been made, it could help solve these problems. Besides, some people stop using ACTs half way through the treatment course because of the side effects they experience. Therefore, participants were of the view that if the single dose treatment was made for the patient to take it once, the side effects could be tolerated since the medication is not repeated.

*“Well, if they can reduce the dosage and it can still treat and kill(cure) the malaria or if they can make it one tablet such that you can just take it once and you don’t take again, I think that will be better. If you take it once and that is all, people can manage and take it and that will be better than where you will take it and get these side effects and yet you have to take the remaining tablets, that is why other people do not normally want to continue and finish.… The other thing is if they can make the injection of it that will also be good because other people do not like taking the tablets because of the taste and the quantity. For me, I prefer taking injection as compared to the tablets”*

***(IDI-32 year old female patient)***

Respondents also suggested that follow-up visits by health workers to the communities to ascertain how people who were given ACTs were using them at home could also improve adherence to malaria treatments.

## Discussion

Currently, there are three recommended ACTs for the treatment of uncomplicated malaria in Ghana. The malaria treatment policy requires that artesunate-amodiaquine, artemether-lumefantrine and dihydroartemisinin-piperaquine be used to treat uncomplicated malaria [5]. This study revealed vast knowledge variation about these ACTs between educated and uneducated participants. The educated participants could mention the name of the ACT given to them at their last malaria episode. Some of the educated participants preferred to use artemether-lumefantrine to treat their malaria. The main reason for their choice of artemether-lumefantrine is that the drug is good and has minimal or no side effects as compared to other ACTs especially artesunate-amodiaquine. This is consistent with a study reported by Mace et al where it was established that ninety percent (90%) of study participants preferred artemether-lumefantrine [12]. The uneducated participants however did not know the type of ACT given at their last malaria episode. This was largely expected given that this could be attributed to inadequate education by health providers. Because of lack of knowledge about the recommended ACTs by the uneducated participants, they could collect any medication given by health providers to treat their malaria. It is possible that this would lead to non-adherence because these patients would be given drugs they will not like using at home.

Adherence to treatment regimens is the extent to which patients use medication as prescribed for them by health care providers [11]. It is therefore very important that patients who are given medications including ACTs use them according to what they have been told by health providers [10]. Unfortunately, our findings show little or inadequate information is being provided to patients with respect to proper use of ACTs and the likely side effects they may experience as a result of using these ACTs. There are various factors that influence patient adherence to the use of ACTs in real life settings. It is reported that the inability of patients with malaria to complete their course of treatment was because of low knowledge on malaria [10]. A recent study in Kenya reported a fifty-three (53%) non-adherence to the use of ACTs [22]. Our findings showed that patients stop using ACTs when they felt that their health condition or illness was better after they have taken the initial dose of the drug. This practice is common among patients especially those who have no formal education to know that they are supposed to use all medications given even when they take the initial doses and feel that there is improvement in their health condition. It is therefore not surprising that participants in our study reported it as the main reason affecting adherence to ACTs use. Some patients would use medications partially or stop the treatment once the symptoms subside and the remaining tablets are kept and used in future [12, 13]. The importance of this practice to the development of resistance to ACTs cannot be overemphasized and must be addressed with the urgency it requires.

This study found that side effects, forgetfulness, inadequate health information by prescribers affected adherence to the use of medications. Various side effects experienced by patients with malaria from the use of ACTs contributed greatly to non-adherence. The inability of some patients with malaria to contain these side effects has made them to stop using these ACTs half way through the treatment regimen [11, 12, 14]. Other patients with malaria do not like taking drugs and because of that they intentionally fail to use ACTs as prescribed [11, 14]. Other important issues revealed in this study were that peer influence, inability to eat heavy food in the morning and the taste of some of the ACTs and the number of tablets they have to take to complete the treatment course impact on patients’ ability to adhere to malaria treatment in the study area.

It is very important for health providers to educate patients on correct use of drugs including ACTs. Based on the views presented by participants in this study, health providers are usually not able to give frequent and enough education to their clients on the need for them to use all medications given even if the condition is better after the patient has taken the initial dosage and the possible side effect they might have experienced. The possible reason for health providers’ inability to provide enough health education to their clients may be as a result of the huge number of clients they usually have to attend to at the Out-Patient Department (OPD) level. Because of the pressure, health providers will usually write on the park on how the patient would use the drug making it difficult for some patients to correctly interpret and use the drug correctly at home especially the uneducated clients.

It is reported that health providers’ inability to provide health education to patients on the correct use of ACTs and the possible side effects associated with the use of these ACTs negatively influenced adherence [11, 14]. It is not therefore surprising that some patients in our study mentioned artemether-lumefantrine as their preferred ACT for treating malaria because of its minimal side effects as compared with artesunate-amodiaquine. A study conducted among patients with malaria who took artemether-lumefantrine revealed sixty percent (60%) adherence [12]. A recent study reported that the use of complex or technical terminologies by prescribers led to wrong use of medications [15]. This has not been mentioned by participants in our study. However, our findings showed that old age is one factor affecting the use of medications. The older people who do not have formal education and therefore cannot read and write are most likely not to adhere to the use of malaria medicines. The issue of recall is a problem for this group of people. However, it is reported that older patients who were given Antihypertensives were more likely to adhere and used the medicines correctly as compared to younger patients [23]. According to the knowledge, attitude and behavior change model, knowledge is a precondition to the intended performance of health related behaviors. Though, attitude could influence the person’s decision, Behavior changes gradually as knowledge accumulates. Therefore, a change in attitude is initiated and over a period of time, changes in attitude accumulate, leading to change in behavior. Behavior change is a gradual process which is achieved through continues motivation [24].

Our study participants suggested intensive continuous health education by health providers and chemical shop owners on the side effects people are likely to experience when they use these ACTs wrongly. This will help address wrong use of medications at home by patients. Evidence exist that patients who received health information were more likely to adhere to malaria treatment than those who have not received health information [10]. Patients with Malaria are not really aware that when they use ACTs wrongly, they are likely to experience not only side effects but contribute to the development of resistance of malaria parasite to ACTs. It is demonstrated that continuous health education on the benefits of using medications given by health providers will reduce the number of people who do not adhere to the use of medications including ACTs [11, 14]. Our study participants suggested a single dose treatment for patients with malaria in order to address issues related to recall and timing problems couple with the number of tablets per day a patient has to take and number of tablets per one malaria episode. The single dose treatment according to the study participants will also address the issue of some patients using malaria medicines half way and stop because of side effects.

## Limitations

Though this study provides information required to increase adherence to the use of malaria treatments, some limitations must be taken into consideration. The interviews were mostly conducted in the main local languages and translated into English. Therefore some words could have lost their original meaning. To help mitigate this, two independent people were made to do the translations and transcription and the transcription were verified. However, given the limitation of such a method, in doing the analysis emphasis was placed on the overarching themes and not specific word choices or phrases.

## Conclusion

Inadequate health information and attitude of some individuals towards the use of medications especially ACTs, side effects and the issue of recall especially among the older people are the main factors influencing adherence to ACTs use in the study area. Despite the fact that some education has been given to patients with malaria at the health facility level on how to use these malaria medications, much efforts still need to be made by health providers in this regard. It is recommended that intensive regular education be carried out at the health facility level on why it is very important for patients to use ACTs correctly. Patients should also be encouraged to use all medications given to them even when there is improvement after the initial dose of the treatment has been taken. There is also the need for health providers to educate their clients on the side effects they are likely to experience from using these ACTs and the need for them to continue and complete the prescribed treatment This will improve on the adherence and individual behavioral factors affecting the use of ACTs and therefore contribute effectively to the reduction in malaria cases.
